# Hydrogen gas with extracorporeal cardiopulmonary resuscitation improves survival after prolonged cardiac arrest in rats

**DOI:** 10.1186/s12967-021-03129-1

**Published:** 2021-11-16

**Authors:** Tai Yin, Lance B. Becker, Rishabh C. Choudhary, Ryosuke Takegawa, Muhammad Shoaib, Koichiro Shinozaki, Yusuke Endo, Koichiro Homma, Daniel M. Rolston, Shuhei Eguchi, Tadashi Ariyoshi, Asami Matsumoto, Kentaro Oka, Motomichi Takahashi, Tomoaki Aoki, Santiago J. Miyara, Mitsuaki Nishikimi, Junichi Sasaki, Junhwan Kim, Ernesto P. Molmenti, Kei Hayashida

**Affiliations:** 1 The Feinstein Institutes for Medical Research, Northwell Health System, 350 Community Drive, Manhasset, NY 11030 USA; 2 Department of Emergency Medicine, North Shore University Hospital, Northwell Health, Manhasset, NY USA; 3 Zucker School of Medicine at Hofstra/Northwell, New York, NY USA; 4 Department of Emergency and Critical Care Medicine, Keio University School of Medicine, Tokyo, Japan; 5 R&D Division, Miyarisan Pharmaceutical Co., Ltd., Saitama, Japan

**Keywords:** Heart arrest, Extracorporeal cardiopulmonary resuscitation, Extracorporeal membrane oxygenation, Hydrogen, Ischemia reperfusion injury

## Abstract

**Background:**

Despite the benefits of extracorporeal cardiopulmonary resuscitation (ECPR) in cohorts of selected patients with cardiac arrest (CA), extracorporeal membrane oxygenation (ECMO) includes an artificial oxygenation membrane and circuits that contact the circulating blood and induce excessive oxidative stress and inflammatory responses, resulting in coagulopathy and endothelial cell damage. There is currently no pharmacological treatment that has been proven to improve outcomes after CA/ECPR. We aimed to test the hypothesis that administration of hydrogen gas (H_2_) combined with ECPR could improve outcomes after CA/ECPR in rats.

**Methods:**

Rats were subjected to 20 min of asphyxial CA and were resuscitated by ECPR. Mechanical ventilation (MV) was initiated at the beginning of ECPR. Animals were randomly assigned to the placebo or H_2_ gas treatment groups. The supplement gas was administered with O_2_ through the ECMO membrane and MV. Survival time, electroencephalography (EEG), brain functional status, and brain tissue oxygenation were measured. Changes in the plasma levels of syndecan-1 (a marker of endothelial damage), multiple cytokines, chemokines, and metabolites were also evaluated.

**Results:**

The survival rate at 4 h was 77.8% (7 out of 9) in the H_2_ group and 22.2% (2 out of 9) in the placebo group. The Kaplan–Meier analysis showed that H_2_ significantly improved the 4 h-survival endpoint (log-rank P = 0.025 vs. placebo). All animals treated with H_2_ regained EEG activity, whereas no recovery was observed in animals treated with placebo. H_2_ therapy markedly improved intra-resuscitation brain tissue oxygenation and prevented an increase in central venous pressure after ECPR. H_2_ attenuated an increase in syndecan-1 levels and enhanced an increase in interleukin-10, vascular endothelial growth factor, and leptin levels after ECPR. Metabolomics analysis identified significant changes at 2 h after CA/ECPR between the two groups, particularly in d-glutamine and d-glutamate metabolism.

**Conclusions:**

H_2_ therapy improved mortality in highly lethal CA rats rescued by ECPR and helped recover brain electrical activity. The underlying mechanism might be linked to protective effects against endothelial damage. Further studies are warranted to elucidate the mechanisms responsible for the beneficial effects of H_2_ on ischemia–reperfusion injury in critically ill patients who require ECMO support.

**Supplementary Information:**

The online version contains supplementary material available at 10.1186/s12967-021-03129-1.

## Introduction

Sudden cardiac arrest (CA) is a major public health problem [1]. Extracorporeal cardiopulmonary resuscitation (ECPR) using a veno-arterial extracorporeal membrane oxygenation (ECMO) device that forces blood circulation using a mechanical pump is often indispensable in patients who lose their pulse for a long period. ECPR can rescue selected patients who do not respond to conventional cardiopulmonary resuscitation (CPR) [2]. Recent systematic reviews and meta-analyses have indicated that ECPR improves survival rates in cohorts of selected patients with out-of-hospital cardiac arrest (OHCA) [3, 4]. However, the long-term survival of such patients with ECPR remains low due to the prolonged ischemia and severe organ damage.

Much evidence has shown that ischemia reperfusion injury including excessive oxidant damage and systemic inflammatory reaction, contributes to mortality and neurological impairment after CA in both humans and animals [5–7]. Despite the benefits of ECPR in the quick restoration of oxygen supply and salvage of ischemic cell death, it is well known that ECMO, which includes an artificial oxygenation membrane and circuits, is associated with oxidative stress and systemic inflammatory reactions, resulting in coagulopathy and endothelial cell damage [8, 9]. Therefore, ECPR can be lifesaving but worsens the undesirable effects of post-CA physiology, thus emphasizing the importance of identifying potential therapeutic targets to improve outcomes in patients with OHCA rescued by ECPR. Further investigations are required to elucidate and target the mechanisms of damage from ECPR.

Inhaled hydrogen (H_2_) selectively reduces cytotoxic reactive oxygen species (ROS) such as hydroxyl radicals (·OH) and peroxynitrite (ONOO^−^) [10]. We have previously demonstrated that inhaled H_2_ attenuates myocardial and brain injury in a rat model of ventricular fibrillation-induced CA [11, 12]. Consequently, clinical pilot studies have shown beneficial effects of H_2_ in patients with acute myocardial infarction [13] and in patients with OHCA who achieved successful return of spontaneous circulation (ROSC) [14]. However, whether H_2_ administration with ECPR can improve outcomes after CA and the precise mechanism responsible for the beneficial effects of H_2_ remain unknown.

Our previous studies have demonstrated that ECPR reliably resuscitates rats after up to 30 min of CA [15, 16]. Additionally, we have demonstrated the foundational metabolic changes in the disease process after prolonged CA in rodents, [17] which were similar to those in human patients [18]. In the current study, we hypothesized that H_2_ administration with ECPR can improve survival in a rat model of highly lethal CA. We also examined whether H_2_ gas protects animals from ECPR-induced endothelial cell damage and inflammatory responses. We also conducted plasma metabolomics analyses to measure global metabolic profiles related to post-CA disease progression in relation to H_2_ treatment.

## Methods

### Animal preparation

Male Sprague–Dawley rats (400–500 g, Charles River) were used in this study. The rats were housed in a rodent facility under a 12-h light/dark cycle and had free access to food and water. All experiments were performed in accordance with the National Institutes of Health guidelines for the use of experimental animals and were approved by the Institutional Animal Care and Use Committee of the Feinstein Institutes for Medical Research.

The rats were intubated, mechanically ventilated, and instrumented under anesthesia with 2% isoflurane as described previously [15–19]. End-tidal carbon dioxide (EtCO_2_) was maintained at 40 ± 5 mmHg during the experiment. The left femoral artery was cannulated (PE-50, Becton Dickinson, Franklin Lakes, NJ) to monitor arterial pressure and the rate of rise in arterial pressure (dP/dt), which are indicators of left ventricular function, [20] and for blood sampling. The left femoral vein was cannulated (PE-50) to monitor central venous pressure (CVP) and for drug administration. The right external jugular vein and right femoral artery were respectively cannulated with a 14 G catheter for venous outflow and with a 20 G catheter for arterial inflow. The esophageal temperature was maintained at 37.0 °C ± 0.5 °C using a thermostatically regulated heating pad and heating lamp throughout the procedure. Blood pressure and needle-probe electrocardiogram monitoring data were recorded and analyzed using a PC-based data acquisition system.

### Rat model of 20-min asphyxial CA and ECPR

This study used a rat model of highly lethal prolonged asphyxia‐induced CA, as reported previously [15–19]. In our previous studies, the survival rate was ~ 20% at 4 h after CA/ECPR [19, 21]. Briefly, after injecting heparin (300 U) and vecuronium (2 mg/kg) via the left femoral vein, asphyxia was induced by stopping mechanical ventilation (MV), and isoflurane was discontinued. A mean arterial pressure (MAP) below 20 mmHg was defined as CA. After 20 min of asphyxia, ECPR was started with the initiation of veno-arterial ECMO flow and resumption of MV. The ECMO circuit consisted of a heat exchanger, an open venous reservoir, a membranous oxygenator (Martin Humbs Engineering, Ingenieurbüro für Bauwesen, German), silicone tubing lines, and a roller pump (MasterFlex, Barrington, IL, USA) primed with 10 mL of Normosol-R (Hospira, Lake Forest, IL), 10 mL of 6% Hespan (B. Braun Medical Inc., Bethlehem, PA, USA) and 0.3 mL of 8.4% sodium bicarbonate (Additional file 1: Fig. S1). As needed, an additional 5 mL of Normosol-R solution and 10 mL of donor blood were added to the venous reservoir to maintain a constant circulating volume. The flow rates reached 130–150 mL/kg/min within 1 min, which approximated the normal cardiac output in the animal. Following ROSC, the flow rate was gradually decreased to 40 mL/kg/min to prevent excessive volume administration. ECMO was performed for 30 min. All animals achieved successful ROSC, and the flow was continued for 30 min after ROSC in all cases. Once the flow was stopped, catheters inserted into the right external jugular vein and right femoral artery were removed, and the surgical wounds were sutured. After ROSC, the animals were ventilated with O_2_ supplemented with or without 2% H_2_ for the first 1 h after ROSC, followed by ventilation with 60% O_2_ for an additional 3 h. Survival time was monitored up to 4 h after CA/ECPR, according to a previous study [19]. For sampling, 0.3 mL of blood was drawn from the left femoral artery catheter at the baseline and at designated times: 0.5, 1, and 2 h after ROSC. Death was defined as a MAP below 30 mmHg, lasting for 5 min.

### Experimental protocol

**Fig. 1:**
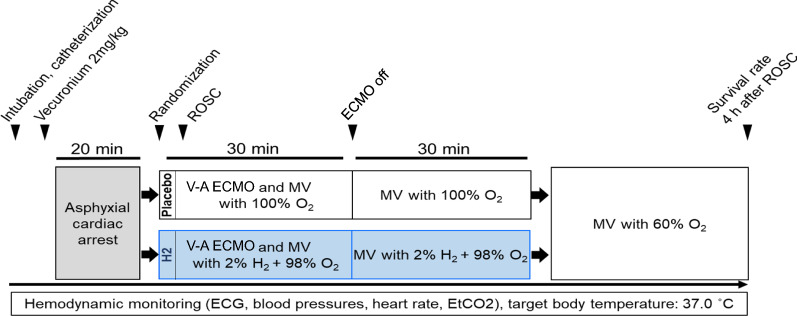
Experimental protocol for extracorporeal cardiopulmonary resuscitation (ECPR) in a rat model of prolonged asphyxia cardiac arrest (CA). V-A ECMO: veno-arterial extracorporeal membrane oxygenation; MV: mechanical ventilation; ROSC: return of spontaneous circulation; ECG: electrocardiogram; EtCO_2_: end-tidal carbon dioxide

When ECPR was started and MV was resumed, animals were randomly assigned to two experimental groups: ECPR with 100% O_2_ (placebo group), and ECPR with 98% O_2_ supplemented with 2% H_2_ (H_2_ group) (Fig. 1). The experimental gases were added to both the membrane oxygenator and ventilator. Rats in both groups were ventilated with the experimental gas for the first 60 min after ROSC. As animals treated with ECPR using 100% or 98% O_2_ had similar survival rates in pilot experiments (data not shown), we used 100% O_2_ as a placebo. Furthermore, this was considered clinically relevant because ECPR for patients with OHCA is usually initiated with 100% O_2_ for the membrane oxygenator and ventilator.

### Assessment of brain function

From our previous studies with varying CA times [19], the toe pinch and corneal reflex were the only stimuli that animals responded to following severe CA. Thus, a lack of response to these stimuli evidenced complete loss of motor responses and a deep unresponsive coma [19]. Therefore, following 20-min asphyxial CA and ECPR, brain function was assessed by the responses to toe pinch and corneal stimulation for 4 h after ROSC.

### Brain tissue oxygen monitoring

The animal’s head was stabilized in a stereotaxic instrument using ear bars. A small burr hole (2 mm) was drilled in the skull at 3 mm lateral and 3 mm posterior to the bregma. PtO_2_ was measured continuously using a Clark type tissue electrode (Integra® Licox® Brain Tissue Oxygen Monitoring, Integra LifeSciences Limited IDA Business and Technology Park, County Offaly, Ireland) inserted at 5 mm. There is no evidence in literature indicating that the Licox® oxygen monitoring analysis is affected by inhaled H_2_.

### Electroencephalogram (EEG) monitoring

In a subgroup of rats subjected to CA and ECPR, we measured EEG for 4 h after ROSC, as reported previously [22]. Briefly, before intubation and cannulation procedures, animals underwent implantation of EEG electrodes bilaterally under isoflurane using a stereotaxic apparatus (Stoelting, USA). Each animal had eight screw electrodes (Plastics One, Roanoke, VA) cortically implanted over the frontal (AP = + 2, L = 2), parietal (AP = − 4, L = 2), occipital (AP = − 6, L = 2), and forelimb regions of the somatosensory cortex (n = 3 per group). The ground electrode was placed over the parasagittal right frontal lobe. The screws were held in place with dental cement. EEGs were recorded using an Intan RHS Stim/Recording 16 channel recording controller during the baseline, asphyxial CA, resuscitation, and after ROSC. Raw EEG signals were used to determine the EEG electrical activity.

### Measurement of plasma syndecan-1

For quantitative determination of soluble syndecan-1 in plasma, a commercially available enzyme-linked immunosorbent assay (ELISA) kit (Novus Biologicals, LLC, CO, USA) was used according to the manufacturer’s instructions. Duplicate measurements were performed for each sample by an investigator blinded to the experiment.

### Multiplex plasma mediator assay

Plasma eotaxin, epidermal growth factor (EGF), fractalkine, interferon (IFN)-γ, interleukin (IL)-1α, IL-1β, IL-2, IL-4, IL-5, IL-6, IL-10, IL-12(p70), IL-13, IL-17A, IL-18, IP-10, growth-regulated oncogenes/keratinocyte chemoattractant (GRO/KC), tumor necrosis factor (TNF)-α, granulocyte-colony stimulating factor (G-CSF), granulocyte–macrophage colony-stimulating factor (GM-CSF), monocyte chemotactic protein (MCP)-1, leptin, lipopolysaccharide-induced CXC chemokine (LIX), macrophage inflammatory protein (MIP)-1α, MIP-2, regulated upon activation normal T cell express sequence (RANTES), and vascular endothelial growth factor (VEGF) levels were determined using the Rat Cytokine/Chemokine Array 27 Plex (Eve Technologies, Calgary, AB, Canada) according to the manufacturer’s protocol. The plasma samples were diluted 1:1 in PBS. Singlet measurements were performed for each sample by an investigator blinded to the experiment, and seven cytokines/chemokines (IL-1α, GM-CSF, IL-2, EGF, IFN-γ, GRO/KC, and MIP-2) were excluded from the analyses because they showed several out of range (OOR) measurements (defined as > 85%) for at least one time point. Values less than the OOR level were replaced with 0.001, as described previously [23].

### Untargeted metabolomics with GC–MS

For metabolomics analysis, we used three groups of plasma: samples obtained before CA induction (pre-CA, n = 8), samples at 2 h post-ROSC in the placebo group (placebo, n = 8), and at 2 h post-ROSC in the H_2_ group (H_2_, n = 8). An investigator blinded to the experiment derivatized the plasma samples for metabolic profiling for GC–MS using a two-step methoximation/silylation derivatization procedure. Plasma sample (50 µL) was mixed with 172 µL of sterile water, 555 µL of methanol, and 222 µL of chloroform by vortex for 20 s, centrifuged at 16,000 rpm at 4 °C for 3 min, and the supernatant was collected. Sterile water (400 µL) was added to the supernatant, and the mixture was centrifuged at 16,000 rpm at 4 °C for 3 min to collect the upper layer of the two-layer separation. The collected layer was evaporated by using a centrifugal concentrator and followed by lyophilization in a freeze dryer. Dried samples were kept drying before derivatization. Myristic acid d-27 in *n*-hexane (0.75 mg/mL, 10 µL) was added to the samples as the derivatization standard. First, the dried samples were methoximated with methoxyamine hydrochloride (40 mg/mL, 10 µL) in anhydrous pyridine at 30 °C for 90 min. Next, the dried samples were silylated with *N*-methyl-*N*-trimethylsilyltrifluoroacetamide using 1% trimethylchlorosilane (TMCS; 90 μL) at 37 °C for 30 min. GC–MS analysis was performed on an Agilent 7890B gas chromatograph connected to an Agilent 5977B MSD (Agilent Technologies UK Ltd.). Samples were injected with an Agilent 7693 autosampler injector into deactivated splitless liners using the FiehnLib settings [24]. Compound identification was performed by comparing the retention time and the mass spectrum with a Fiehn metabolomics mass spectrum library [24]. Peaks with a similarity index higher than 60% were assigned compound names, whereas those with less than 60% similarity were listed as unknown metabolites. The chromatograms were subjected to noise reduction prior to peak area integration. Any known artificial peaks, such as those due to noise, column bleeding, and the *N*-methyl-*N*-trimethylsilyltrifluoroacetamide derivatization procedure, were excluded from the data set. The integrated peak areas of multiple derivative peaks belonging to the same compound were summed and considered as a single compound. The relative peak area of each compound was calculated as the response after integrating the peak areas of the compounds.

### Metabolomics data analysis

In total, 189 metabolites across the groups were detected using GC–MS. Of these, 76 metabolites in samples with a coefficient of variation (CV) value > 30% were rejected, which is acceptable for biomarker discovery [25]. Of the 113 remaining metabolites, variables with missing data (> 50% as threshold) were removed. Thereafter, the missing variables were replaced with the limit of detection (1/5 of the minimum positive value for each variable), as per the default setting of MetaboAnalyst [26]. Finally, a total of 53 metabolites were used for the study.

Principal component analysis (PCA) was used to gain an overview of all the samples and to identify the possible outliers. To visualize the changing patterns in metabolites for facilitate comparison among the three groups, a heatmap was generated by hierarchical clustering of plasma metabolites. Partial least squares-discriminant analysis (PLS-DA) identifies metabolites that carry the greatest group-separating information, as represented by the first latent variable. Using supervised machine learning, PLS-DA examines the discriminative capacity of high-dimensional and highly correlated data (metabolomics) and the relative importance of each feature within the dataset (i.e., each metabolite in the present study) [27]. PLS-DA was thus used to identify significantly changed metabolites among different groups (variable importance in the projection, VIP > 1 as a mean of all components). The models were refined by VIP selection to maximize Q2 (i.e., the cross-validated R2). A volcano plot was also generated to screen features with statistical significance (p < 0.05) and fold change (FC) > 1.2 or < 0.8. Finally, based on the VIP selection obtained using PLS-DA of the placebo and H_2_ groups, metabolite set enrichment analysis (MSEA) was conducted to evaluate the impact of individual metabolite alterations on different metabolic pathways. The MSEA was declared positive (i.e., differentially regulated) if it had a false discovery rate (FDR) < 0.05. The PCA, PLS-DA, volcano plots, heatmap, and MSEA were all performed with MetaboAnalyst (v5.0) using normalized data (auto-scaling feature and log transformation).

### Statistical analysis

Data for continuous variables are presented as mean ± standard error of the mean (SEM). Categorical data are presented as counts with frequencies. An unpaired two-tailed Student’s *t*-test or Mann–Whitney U test was used to compare two independent groups, as appropriate for continuous variables. For normally distributed data, two-way analysis of variance (ANOVA) with or without repeated measures followed by Sidak’s correction for post-hoc comparisons was used as appropriate. For non-normally distributed data, the Friedman test followed by Dunn’s multiple comparisons was used for post-hoc comparisons. Survival rates were estimated using the Kaplan–Meier method, and the log-rank test was used to compare the survival curves between groups. Statistical significance was considered at P < 0.05. GraphPad Prism 7.05 (GraphPad Software Inc., La Jolla, CA, USA) and SPSS (version 25.0; SPSS Inc., Chicago, IL, USA) were used for statistical analyses.

## Results

### H_2_ with ECPR improved survival outcomes after CA

**Fig. 2:**
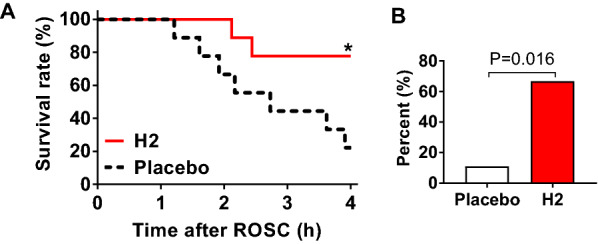
H_2_ improved survival and brain recovery after CA/ECPR. **A** Survival rates during the first 4 h after CA. *log-rank P = 0.025 vs. placebo group. **B** Percentage of animals exhibiting responses to either or both the stimuli. n = 9 per group

The baseline variables (body weight, MAP, heart rate, CVP, body temperature, arterial dP/dt(max), dP/dt(min), and EtCO_2_) were the same in each group (Additional file 2: Table S1). There was no difference between each group in the time from ECPR initiation to ROSC, duration of pulseless electrical activity, or the parameters during ECPR except for the CVP at 10 min after ECPR initiation (CVP, placebo vs. H_2_: 5.7 ± 0.8 vs. 3.1 ± 0.8 mmHg, P = 0.036, Additional file 2: Table S2). There were no differences in the arterial lactate, pH, PaO_2_, PaCO_2_, base excess, or HCO_3_ levels for the first 2 h after CA between the groups (Additional file 1: Fig. S2). The survival rate at 4 h after CA/ECPR was 77.8% (7 out of 9) in the H_2_ group and 22.2% (2 out of 9) in the placebo group. Kaplan–Meier analysis showed that H_2_ markedly improved the survival time after prolonged CA (log-rank P = 0.025 vs. placebo, Fig. 2A).

**Fig. 3:**
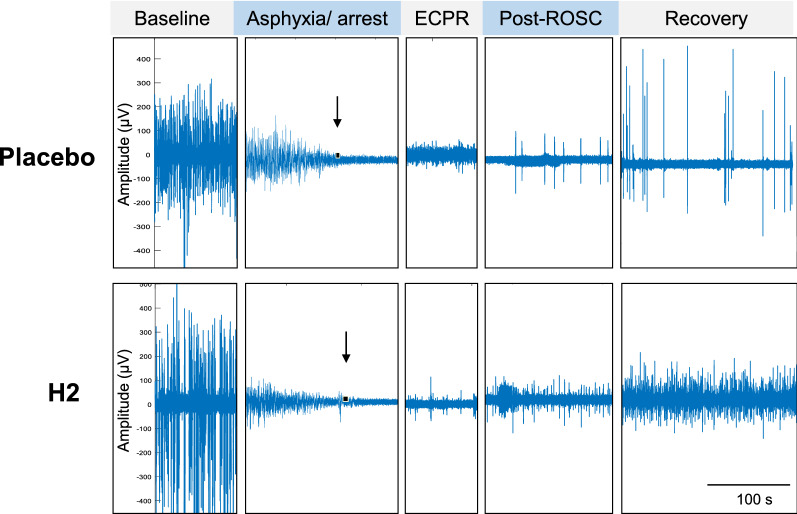
Representative electroencephalogram (EEG) wave forms showing brain electrical recovery after CA/ECPR. The bar indicates 100 s. Arrows indicate the time point of EEG disappearance

Following prolonged CA and ECPR, brain function was assessed based on the response to toe pinch and corneal stimulation. During the experiment, none of the animals showed a response to toe pinch, and one out of nine rats had corneal reflex in the placebo group. In the H_2_ group, two of nine animals responded to a toe pinch, and four rats showed corneal reflex. In total, the percentage of animals exhibiting either or both responses was significantly higher in the H_2_ group than in the placebo group (66.7% vs. 11.1%, P = 0.016 by Chi-square test, Fig. 2B). The representative EEG waveforms in both groups are shown in Fig. 3. In all animals, EEG amplitudes disappeared at 51 ± 15 s after CA. After ROSC by ECPR, none of the animals treated with the placebo (0 out of 3) regained continuous EEG for the first 4 h after ROSC, whereas animals treated with H_2_ (3 out of 3) regained continuous EEG at 47 ± 9.9 min after ROSC, suggesting the beneficial effects of H_2_ on post-CA brain electrical recovery. These observations suggest that H_2_ administration along with ECPR improved the survival outcomes and brain recovery after CA and ECPR.

### H_2_ improved brain oxygenation during ECPR

**Fig. 4:**
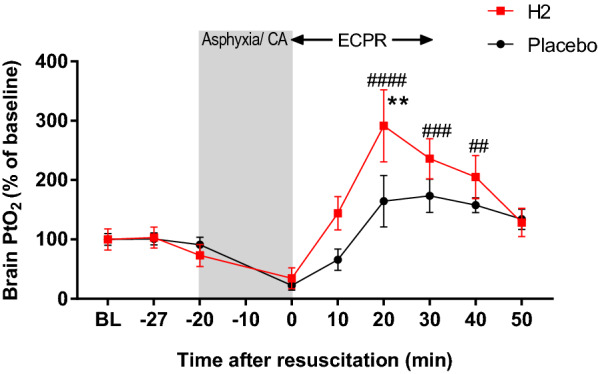
Changes in brain tissue oxygenation (PtO_2_, % change from baseline) during CA and ECPR with and without H_2_. n = 7 per group (two-way repeated-measures ANOVA followed by Sidak’s correction, F = 2.51). **P = 0.009 vs. the placebo, ^##^P = 0.007, ^###^P = 0.0002, ^####^P < 0.0001 vs. the baseline in the H_2_ group

To determine the impact of H_2_ on brain recovery after ECPR, we measured tissue oxygenation with PtO_2_ during ECPR in the brains of animals subjected to prolonged CA and resuscitated with placebo or H_2_. After 20 min of asphyxial CA, the brain PtO_2_ of all animals markedly decreased to 28.6% ± 6.0% of baseline. In the placebo group, ECPR with 100% O_2_ restored the PtO_2_ levels at around the baseline level, which was ventilated with 30% O_2_. However, PtO_2_ for the first 50 min after ECPR initiation did not differ from the baseline level in the placebo group. In contrast, PtO_2_ in H_2_-treated animals increased strikingly to more than 200% of the baseline at 20–40 min after ECPR initiation. PtO_2_ recovery at 20 min after ECPR initiation was significantly higher in the H_2_ group than in the placebo group (291% vs. 164% of baseline, P = 0.009) (Fig. 4).

### H_2_ attenuated the elevated CVP observed early after CA and ECPR

**Fig. 5:**
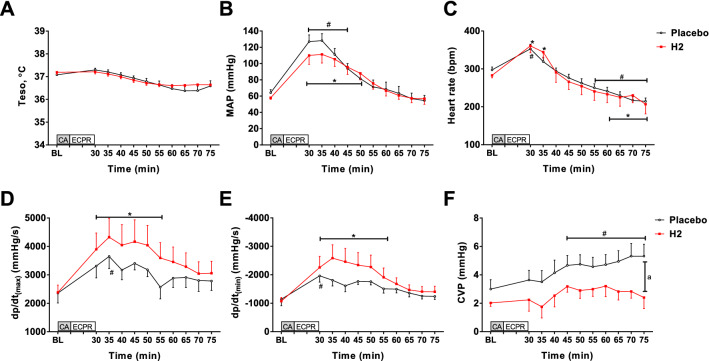
Changes in the **A** esophageal temperature (Teso), **B** mean arterial pressure (MAP), **C** heart rate (HR), **D** dP/dt_max_, **E** dP/dt_min_, and **F** central venous pressure (CVP) during CA and ECPR. Two-way repeated-measures ANOVA followed by Sidak’s correction for post-hoc comparisons were used. F values for Teso, MAP, HR, dP/dt_max_, dP/dt_min_, and CVP were 1.89, 0.77, 0.58, 0.56, 1.18, and 1.15, respectively. Data are presented as the mean ± SEM. *BL* baseline, *CA* cardiac arrest; *ECMO* extracorporeal membrane oxygenation. ^#^P < 0.05 vs. the baseline in the placebo group; *P < 0.05 vs. the baseline in the H_2_ group; ^a^P < 0.05 between groups

We examined the impact of H_2_ administration on hemodynamic parameters during the post-CA reperfusion period. In both groups, MAP increased within approximately 50 min after ROSC, and HR decreased approximately 1 h after ROSC. During the first 75 min after CA, there was no difference between the groups in body temperature, MAP, or HR (Fig. 5A–C). The H_2_ group exhibited markedly higher dP/dt(max) and lower dP/dt(min) early after ROSC compared with the baseline (Fig. 5D, E). Notably, there was a significant difference with respect to CVP. In the placebo group, CVP gradually increased after CA/ECPR. However, in the H_2_ group, CVP was maintained for the first 75 min, and H_2_-treated animals exhibited markedly lower CVP than the placebo group at 75 min after CA (P = 0.038, Fig. 5F). These observations suggest that H_2_ with ECPR improves myocardial contractility and thus attenuates central venous congestion early after ECPR.

### H_2_ attenuated excessive shedding of endothelial glycocalyx in plasma and enhanced plasma IL-10, leptin, and VEGF levels after CA/ECPR

**Fig. 6:**
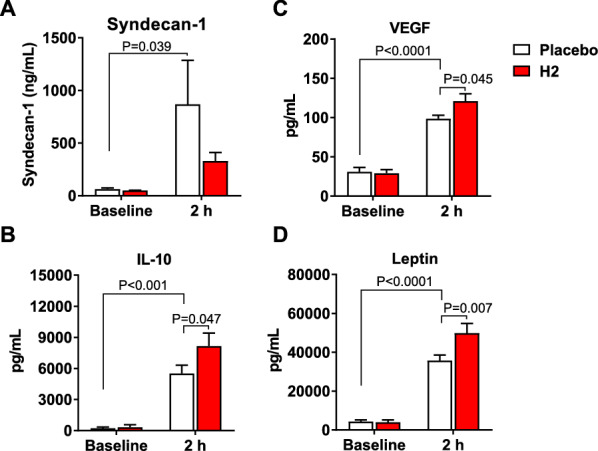
Effects of H_2_ on plasma mediators after CA/ECPR. Plasma levels of **A** syndecan-1, **B** interleukin (IL)-10, **C** vascular endothelial growth factor (VEGF), and **D** leptin at the baseline and at 2 h post-ECPR in animals treated with the placebo and H_2_. n = 8 per group. Two-way repeated-measures ANOVA followed by Sidak’s correction for post-hoc comparisons were used. F values for Syndecan-1, IL-10, VEGF, and leptin were 1.47, 2.94, 3.97, and 6.76, respectively. Data are presented as the mean ± SEM

To determine the mechanisms associated with the protective effects of H_2_, we measured plasma syndecan-1 levels at the baseline and 120 min after CA with or without H_2_. Cardiac arrest and ECPR led to a considerable increase in plasma syndecan-1 levels at 120 min. H_2_ with ECPR abated the increase in plasma syndecan-1 levels (Fig. 6A), indicating that the protective effects of H_2_ on the outcomes are associated with an inhibitory effect on the release of endothelial glycocalyx shedding into the blood. We also examined plasma cytokine and chemokine levels at 2 h post-ROSC with and without H_2_. Cardiac arrest and ECPR markedly increased the plasma levels of IL-10, VEGF, leptin, TNF-α, IL-1β, IL-6, fractalkine, IL-5, IL-18, IP-10, IL-4, eotaxin, MIP-1α, IL-17α, IL-12, RANTES, G-CSF, LIX, and MCP-1. However, H_2_ gas administration at this time point did not affect the plasma levels of TNF-α, IL-1β, IL-6, fractalkine, IL-5, IL-18, IP-10, IL-4, eotaxin, MIP-1α, IL-17α, IL-12, RANTES, G-CSF, LIX, and MCP-1 (Fig. 6 and Additional file 1: Fig. S3), whereas the plasma levels of IL-10, VEGF, and leptin were increased by H_2_ (Fig. 6B–D).

### Changes in plasma metabolites after CA and ECPR

**Fig. 7:**
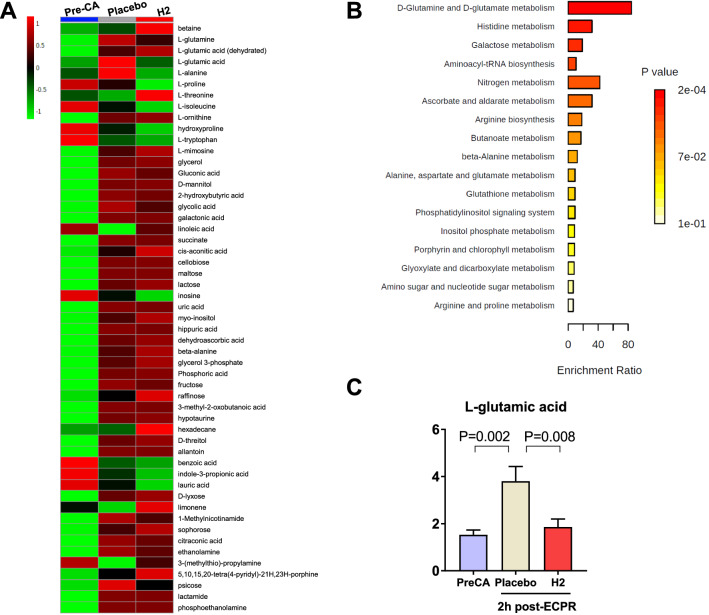
Metabolomic analysis. **A** Heat map comparisons of differential metabolite alterations between groups. The heatmap was generated with MetaboAnalyst 5.0 using normalized data (log transformation, Auto scaling) and Euclidean distance. **B** Enrichment analysis. Metabolite set enrichment analysis (MSEA) was conducted to evaluate the impact of individual metabolite alterations between the placebo and H_2_ groups. MSEA identified d-glutamine and d-glutamate metabolism (FDR = 1.62 × 10^−2^) as significantly discriminated among the groups. **C** Changes in plasma l-glutamic acid after CA/ECPR with and without H_2_ (one-way repeated-measures ANOVA followed by Sidak’s correction, F = 8.33)

Unbiased PCA analysis showed metabolites from pre-CA and post-CA samples with unique and distinct clusters, confirming a sufficient sample size to conduct further analyses (Additional file 1: Fig. S4). PLS-DA also confirmed this distinct clustering of metabolites among the three groups (Additional file 1: Fig. S5). The heatmap of metabolites of the relative changes among the three groups is shown as a visual representation in Fig. 7A. The metabolomic response post-CA was then assessed by comparing the pre-CA and placebo groups. Based on PLS-DA and volcano plot analysis, 27 significantly altered metabolites were identified in the placebo group compared to the pre-CA group; the detailed information of VIP, log2(FC), p values are listed in Additional file 2: Table S3. Next, to determine the impact of H_2_ on metabolites after CA/ECPR, we assessed the metabolomic response to post-CA between the placebo and H_2_ treatment groups. In the H_2_ group, six significantly altered metabolites (myo-inositol, l-histidine, raffinose, mannitol, l-glutamic acid, and *N*-acetylneuraminic acid) were identified compared to the placebo group (Additional file 2: Table S3). Based on PLS-DA analysis between the placebo and H_2_ groups, we conducted MSEA. Additional file 2: Table S4 shows the results from the MSEA. MSEA identified d-glutamine and d-glutamate metabolism (FDR = 1.62 × 10^−2^) to be significantly altered among the groups (Fig. 7B).

## Discussion

The present study demonstrated the beneficial effects of H_2_ when used in combination with ECPR on short-term outcomes in a highly lethal rat model of CA. H_2_-treated animals showed improved survival time, with improvements in the functional status and electrical recovery of the brain after CA/ECPR. The beneficial effects of H_2_ were associated with increased PtO_2_ in the brain during ECPR, and a decrease in plasma syndecan-1 levels, concurrent with the enhancement of plasma mediators after CA. Moreover, these findings provide the unique evidence of metabolic derangements, specifically a shift in d-glutamine and d-glutamate metabolism, in experimental CA/ECPR treated with H_2_.

It has been shown that inhalation of 1% to 4% H_2_ can reduce infarct size in rat models of acute cerebral and coronary artery occlusion, with 2% H_2_ being the most effective [10]. In addition. inhalation of 2% H_2_ with 98% O_2_ starting at the beginning of CPR and administered for 2 h after ROSC significantly improves the outcomes in a rat model of CA with ventricular fibrillation [11]. Based on these reports, we chose 2% H_2_ and 98% O_2_ as an experimental gas so that the H_2_ gas concentration did not exceed an explosive threshold of 4% at room temperature.

Hypoxic-ischemia brain injury is common in patients who receive ECPR [28]. Although the pathophysiology of brain injury during ECMO is not fully understood, inadequate cerebral oxygenation, cerebral blood flow (CBF) alterations, and abrupt PaCO_2_ changes may play a critical role [29]. In our rat model of CA and ECPR, arterial lactate, PaO_2_, and pH, but not PaCO_2_, were markedly changed relative to the baseline upon initiating ECPR, but there was no difference in these parameters between post-CA animals with and without H_2_. During ECPR, H_2_-treated animals exhibited markedly higher O_2_ levels in the brain tissue compared to placebo-treated animals. Previous studies have shown that a higher occurrence of cerebral oximetry desaturation during V-A ECMO was independently associated with mortality in patients who underwent ECMO [30, 31]. Together, these observations indicate that the salutary impact of H_2_ administration on survival and EEG recovery could be linked with improved brain tissue oxygenation during ECPR. In addition, as CBF is dependent on constantly receiving a significant proportion of the cardiac output, improved mortality in the H_2_ group relative to the placebo group could be, at least partially, a consequence of better cerebral perfusion, because the parameters (dP/dt max and min, CVP) after ECPR tended to improve in the H_2_ group.

Our results clearly demonstrate, for the first time, that prolonged CA and subsequent ECPR led to a considerable increase in plasma syndecan-1 levels at 2 h after ROSC in rats. Previous experimental models have also demonstrated that ischemia reperfusion damages the endothelial glycocalyx [32–34], leading to the release of glycocalyx components such as syndecan-1 and heparan sulphate [34]. Further, initiation of extracorporeal circulatory support is associated with excessive ROS and a complex innate immune response [35, 36]. leading to endothelial injury [9]. Glycocalyx degradation is activated by ROS and pro-inflammatory cytokines, resulting in blood–brain barrier (BBB) leakage, brain edema, and poor neurologic outcomes after CA/CPR in rats [37]. Preservation of the glycocalyx by hydrocortisone reduces BBB permeability and gene transcription-protein synthesis and inflammation, thus improving the neurologic outcomes after CA/CPR [37]. A previous study has also shown that H_2_ suppresses the TNF-α release and endothelial glycocalyx degradation, thus preventing endothelial damage after hemorrhagic shock in rats [38]. Based on the well-established anti-oxidant property of H_2_ [10–12, 38], we hypothesized that H_2_ is sufficient to attenuate the endothelial glycocalyx degradation caused by CA/ECPR. We also asked whether H_2_ exerts its therapeutic effect by suppressing the systemic inflammatory response after ECPR. In the present study, H_2_ was found to abate the excessive release of syndecan-1 into plasma after CA and ECPR, consistent with the previous study [38]. Unexpectedly, H_2_ did not affect the plasma levels of most pro-inflammatory cytokines/chemokines after ECPR. However, the beneficial effects of H_2_ were associated with an exaggerated increase in the IL-10, VEGF, and leptin levels.

Many preclinical and clinical studies have demonstrated an association between cytokines and glycocalyx degradation biomarkers [39–42]. IL-10, a well-established anti-inflammatory cytokine, can block NF-κB activity, thereby decreasing the expression of cell adhesion molecules on the endothelial cell surface and thus inhibit leukocyte transmigration [43, 44]. Our results thus indicate that the beneficial effects of H_2_ with ECPR are associated with an enhanced anti-inflammatory response. The precise role of VEGF after CA and ECPR is not fully understood; however, VEGF is considered an endothelial survival factor that prevents microvascular apoptotic cell loss in vitro [45]. Notably, the relationship between VEGF levels and mortality in critical illness is discordant in different studies [46–48]. Leptin is generally considered a rapid stress mediator after injuries and has been found to exert neuroprotective effects in a mouse model of transient focal cerebral ischemia [49]. Taken together, higher plasma IL-10, VEGF, and leptin concentrations shortly after reperfusion may provide beneficial effects on cerebrovascular damage after CA/ECPR, but their detailed relationship with mortality needs to be evaluated and confirmed in further studies.

Metabolomics analysis is an emerging technology that enables rapid and simultaneous measurement of several small functional molecules. Previously, we have demonstrated that several metabolic pathways were altered after CA and after 30 min of ECPR in multiple organs including the brain, heart, and kidney in rats [17]. In addition, the metabolic milieu of the plasma in human patients with OHCA was substantially similar to that in our rodent model of highly lethal CA and ECPR [18]. Based on these observations, the current study was designed to determine whether there were significant differences in metabolomic profiles during the acute phase of post-resuscitation care (i.e., 2 h post-ROSC) between animals post-ECPR with and without H_2_. We observed that d-glutamine and d-glutamate metabolism was the only impacted pathway that differentiated between the placebo and H_2_-treated animals. Glutamate is the most abundant excitatory neurotransmitter in the brain and is neurotoxic when present in excessive amounts extracellularly [50]. In our study, plasma l-glutamic acid was markedly increased by CA and following ECPR compared to the pre-CA levels, whereas H_2_ markedly attenuated the increased l-glutamate at 2 h post-ROSC (Fig. 7C). Although we could not identify the precise H_2_ internalization mechanisms in the current metabolomics analysis, the effect of H_2_ on functional outcomes after CA/ECPR might be explained by the reduction of neurotoxicity from glutamic acid. However, further studies are required to uncover the mechanism underlying the effects of H_2_ on ischemia reperfusion injury.

Our study had several limitations. First, the rodent brain has properties different from those of the complex human brain. Thus, findings analogous to those from the rodent model are yet to be demonstrated in large animal and clinical studies. Second, H_2_ administration was not blinded, whereas other experiments such as multiplex measurements and metabolomic analyses in this study, were conducted in a fully blinded manner. Third, because the neurological outcome was assessed only based on the responses to stimuli and EEG monitoring, further studies are thus needed to determine whether H_2_ directly confers protection to the brain after ECPR. Fourth, we used only 4 h of recovery from CA/ECPR according to a previous study [19]; thus, the long-term efficacy of H_2_ as an adjunct to ECPR on brain injury needs to be verified in the appropriate experimental models. Fifth, we used only male animals in our study and therefore the results may not yield a complete understanding of the role of ECPR and H_2_ treatment. Finally, the experimental design does not allow conclusions to be drawn regarding the best concentrations or the best time window of H_2_ therapy for CA and ECPR.

## Conclusions

In conclusion, we have demonstrated for the first time that combined therapy with ECPR and H_2_ improved intra-resuscitation brain oxygenation and survival time in a rat model of highly lethal CA. Our data suggest that H_2_ combined with ECPR may be an innovative therapeutic strategy for patients with CA who require ECPR. Further studies are warranted to elucidate the mechanisms responsible for the beneficial effects of H_2_ on ischemia–reperfusion injury in critically ill patients who require ECMO support.

## Supplementary Information

**Additional file 1: Figure S1.** Scheme of the rat ECPR model using cardiopulmonary bypass and an extracorporeal membrane. The experimental gas (placebo or H_2_) was administered via a ventilator and an extracorporeal oxygenator. 1, capnograph; 2, oxygen saturation and hematocrit; 3, pump speed (mL/min); 4, temperature, PO_2_, and system pressure of arterial return; 5, rectal temperature; 6, arterial pressure and central venous pressure and arterial blood gas analysis; 7; electrocardiogram; 8; esophageal temperature. **Figure S2.** Changes in arterial lactate, PaO_2_, PaCO_2_, pH, base excess, and HCO_3_^−^ during CA and ECPR. Data are presented as the mean ± SEM. BL, baseline. *P < 0.05 vs. baseline. **Figure S3.** Changes in the plasma mediators after CA and ECPR. Plasma levels of interleukin (IL)-1β, macrophage inflammatory protein (MIP)-1α, IL-4, IL-5, IL-6, IL-12 (P70), IL-13, IL-17α, IL-18, tumor necrosis factor (TNF)-α, fractalkine, granulocyte colony stimulating factor (G-CSF), interferon-γ-inducible protein (IP)-10, monocyte chemotactic protein (MCP)-1, eotaxin, lipopolysaccharide-induced CXC chemokine (LIX), and RANTES at the baseline and at 2 h after ECPR in animals treated with the placebo and H_2_. n = 8 per group. Data are presented as mean ± SEM. **Figure S4.** Principal component analysis (PCA). (A) PCA of metabolites in plasma samples from the pre-cardiac arrest (CA), placebo (2 h post-ECPR), and H_2_ (2 h post-ECPR) groups. (B) Loading plot of metabolites. **Figure S5.** Partial least squares discriminant analysis (PLS-DA). (A) PLS-DA confirmed the distinct clustering of metabolites among the three groups. (B) Loading plot of the metabolites.

**Additional file 2: Table S1.** Group characteristics before resuscitation in rats of placebo and H2 group. **Table S2.** Physiological and interventional variables during ECPR in rats of placebo and H2 group. **Table S3.** Detailed information of perturbed metabolites (VIP > 1.0) among the groups (Placebo vs. Pre-CA, or H2 vs. Placebo). **Table S4.** Result from metabolite set enrichment analysis (MSEA).

## Data Availability

The datasets used and/or analyzed during the current study are available from the corresponding author on reasonable request.

## Abbreviations

BBB: Blood–brain barrier
CA: Cardiac arrest
CBF: Cerebral blood flow
CPR: Cardiopulmonary resuscitation
CV: Coefficient of variation
CVP: Central venous pressure
dP/dt: Rate of rise in arterial pressure
ECMO: Extracorporeal membrane oxygenation
ECPR: Extracorporeal cardiopulmonary resuscitation
EEG: Electroencephalogram
EGF: Epidermal growth factor
EtCO_2_: End-tidal carbon dioxide
FDR: False discovery rate
H_2_: Hydrogen
IL: Interleukin
IFN: Interferon
G-CSF: Granulocyte-colony stimulating factor
GM-CSF: Granulocyte-macrophage colony-stimulating factor
GRO/KC: Growth-regulated oncogenes/keratinocyte chemoattractant
LIX: Lipopolysaccharide-induced CXC chemokine
MAP: Mean arterial pressure
MCP: Monocyte chemotactic protein
MSEA: Metabolite set enrichment analysis
MIP: Macrophage inflammatory protein
OOR: Out of range
PCA: Principal component analysis
PLS-DA: Partial least squares-discriminant analysis
RANTES: Regulated upon activation normal T cell express sequence
ROS: Reactive oxygen species
ROSC: Return of spontaneous circulation
SEM: Standard error of the mean
TNF: Tumor necrosis factor
VEGF: Vascular endothelial growth factor
VIP: Variable importance in the projection
