# Supplementary figures and images for: Hydrogen gas with extracorporeal cardiopulmonary resuscitation improves survival after prolonged cardiac arrest in rats
Source: J Transl Med. 2021 Nov 16;19:462. doi: 10.1186/s12967-021-03129-1 (PMC8594155; doi:10.1186/s12967-021-03129-1)

Figure S1

- Placebo (100%O<sub>2</sub>) or
- H<sub>2</sub> (2%H<sub>2</sub> + 98%O<sub>2</sub>)

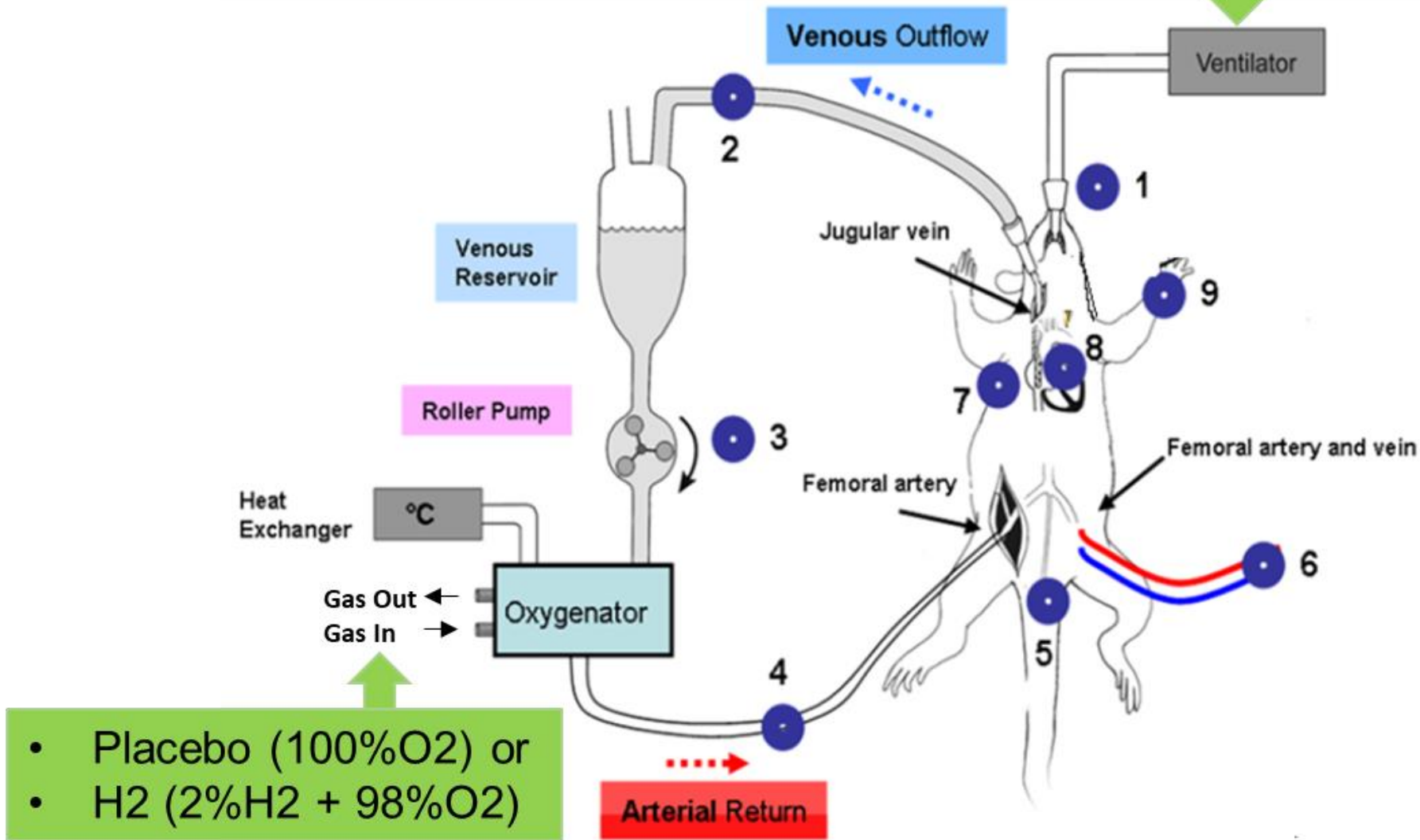

Figure S2

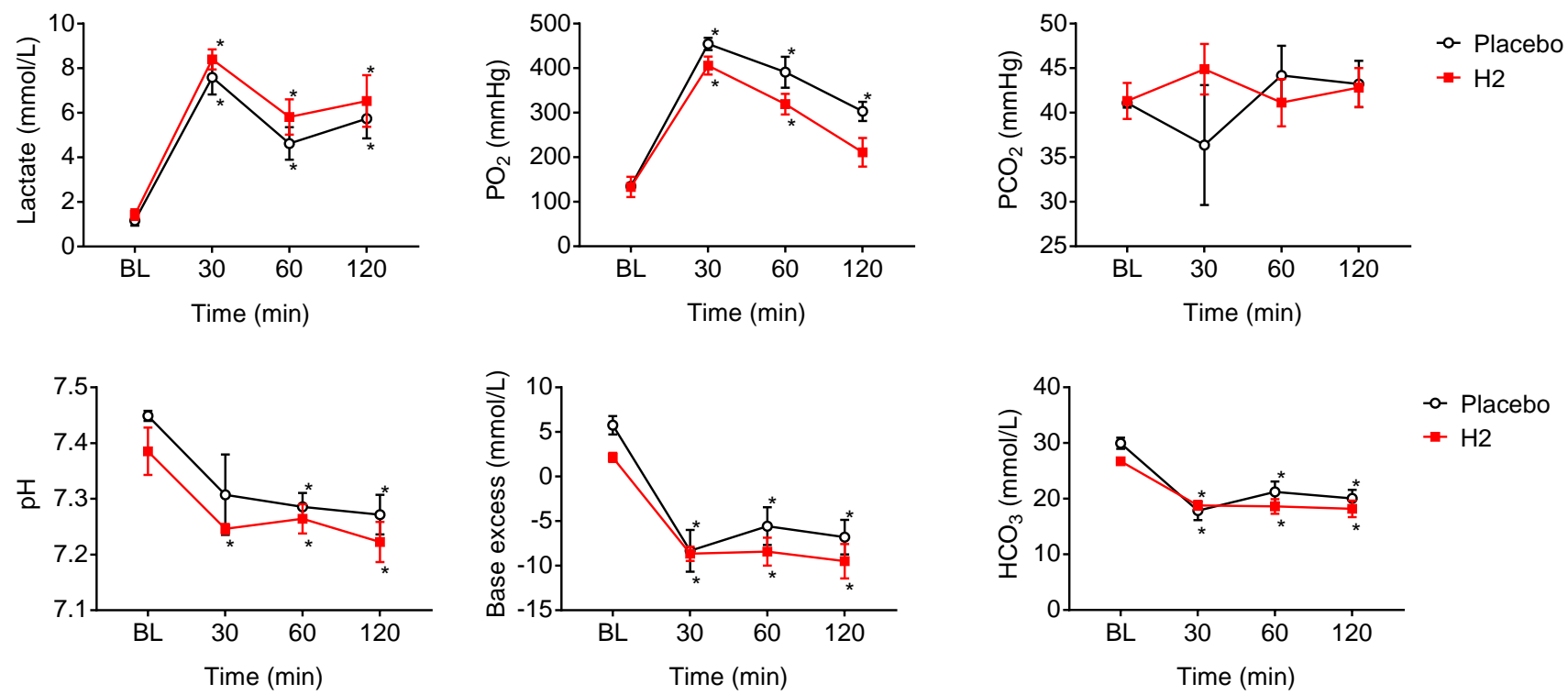

Figure S3

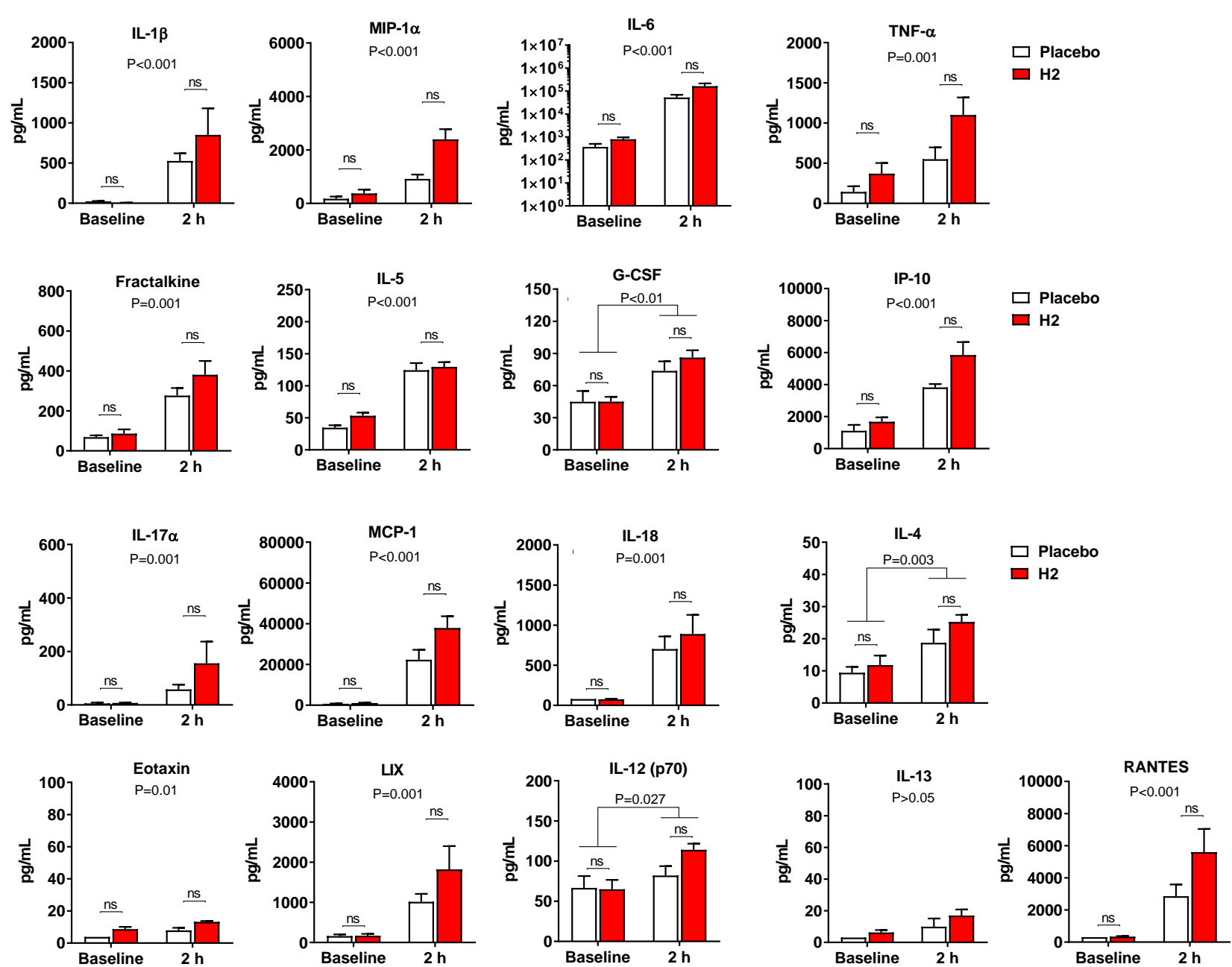

Figure S4

A

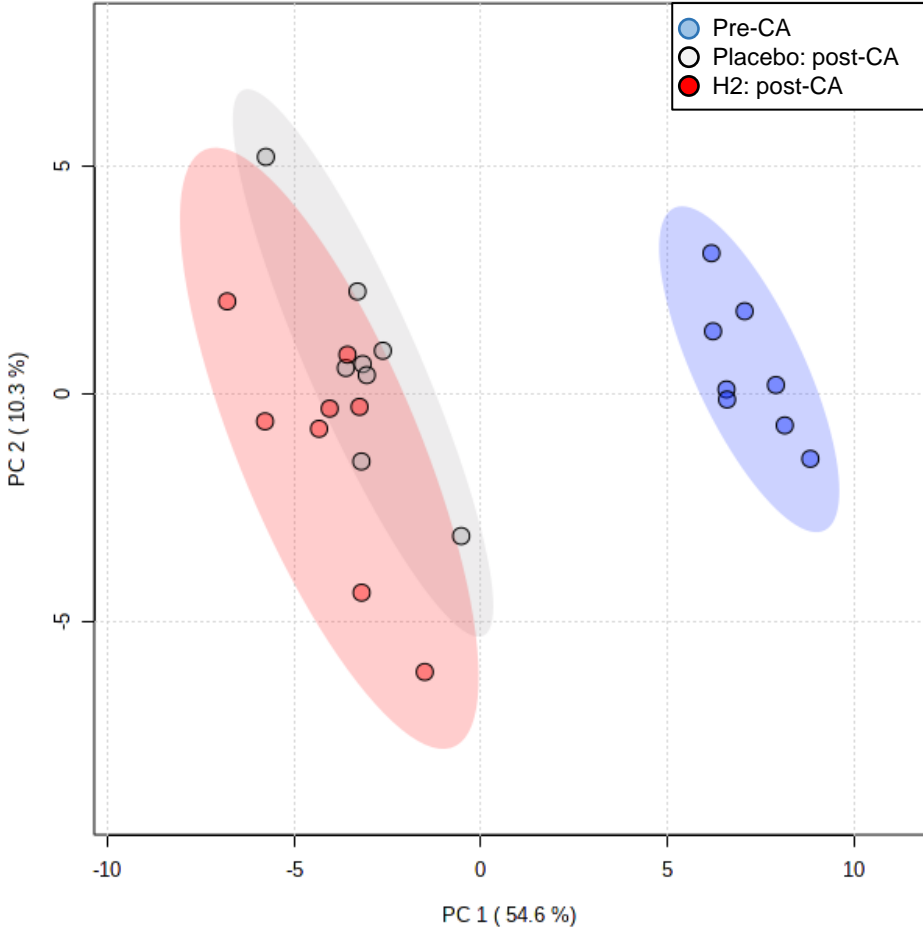

B

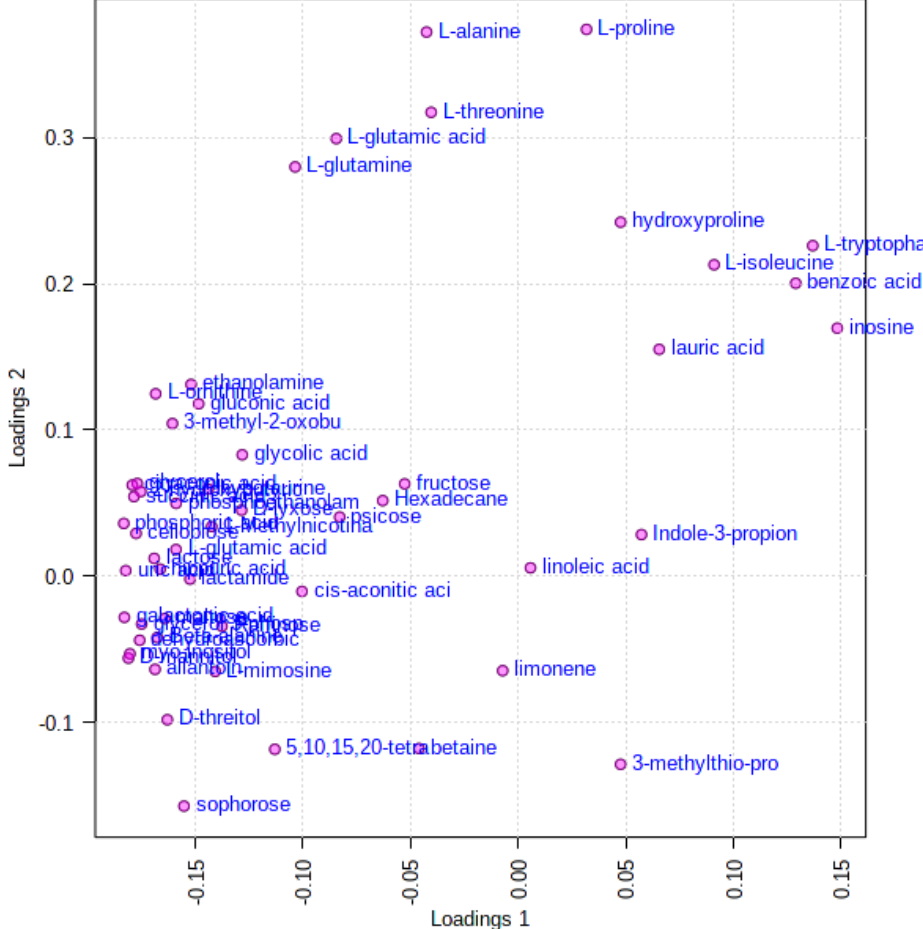

**A**

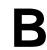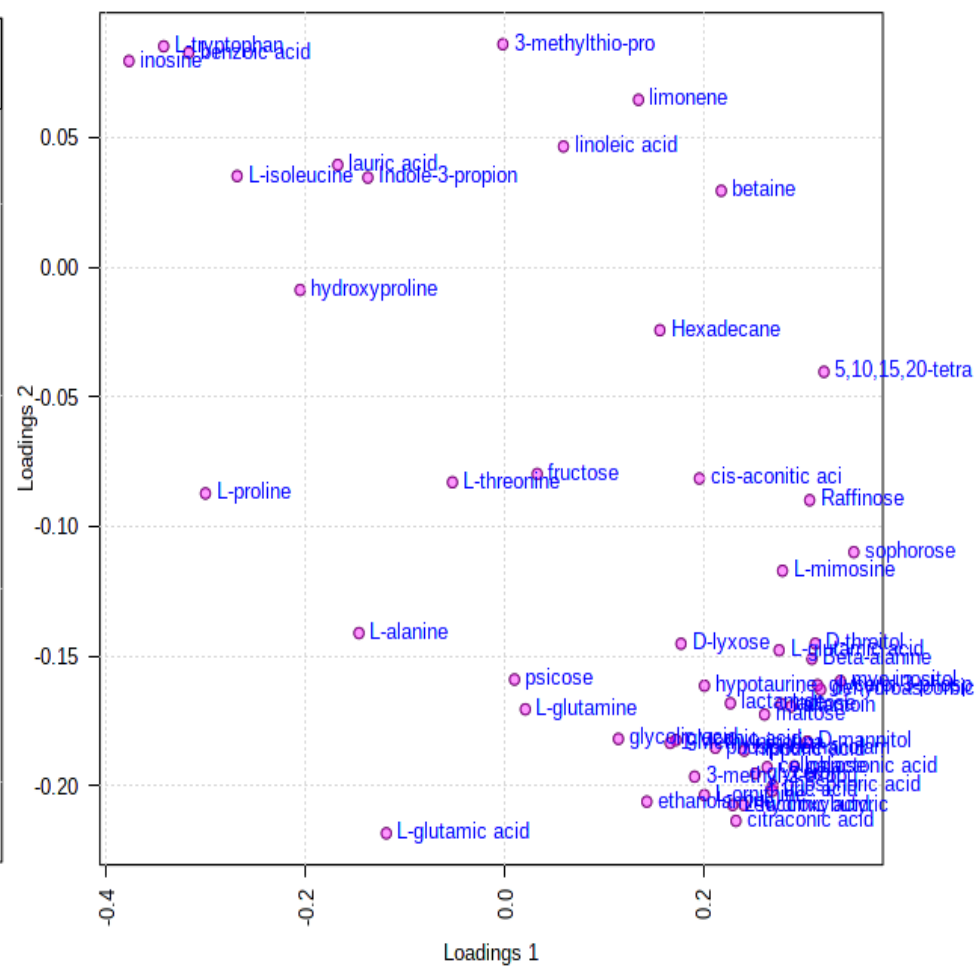

Supplement: Supplementary file 1 — Additional file 1: Figure S1. Scheme of the rat ECPR model using cardiopulmonary bypass and an extracorporeal membrane. The experimental gas (placebo or H2) was administered via a ventilator and an extracorporeal oxygenator. 1, capnograph; 2, oxygen saturation and hematocrit; 3, pump speed (mL/min); 4, temperature, PO2, and system pressure of arterial return; 5, rectal temperature; 6, arterial pressure and central venous pressure and arterial blood gas analysis; 7; electrocardiogram; 8; esophageal temperature. Figure S2. Changes in arterial lactate, PaO2, PaCO2, pH, base excess, and HCO3− during CA and ECPR. Data are presented as the mean ± SEM. BL, baseline. *P < 0.05 vs. baseline. Figure S3. Changes in the plasma mediators after CA and ECPR. Plasma levels of interleukin (IL)-1β, macrophage inflammatory protein (MIP)-1α, IL-4, IL-5, IL-6, IL-12 (P70), IL-13, IL-17α, IL-18, tumor necrosis factor (TNF)-α, fractalkine, granulocyte colony stimulating factor (G-CSF), interferon-γ-inducible protein (IP)-10, monocyte chemotactic protein (MCP)-1, eotaxin, lipopolysaccharide-induced CXC chemokine (LIX), and RANTES at the baseline and at 2 h after ECPR in animals treated with the placebo and H2. n = 8 per group. Data are presented as mean ± SEM. Figure S4. Principal component analysis (PCA). (A) PCA of metabolites in plasma samples from the pre-cardiac arrest (CA), placebo (2 h post-ECPR), and H2 (2 h post-ECPR) groups. (B) Loading plot of metabolites. Figure S5. Partial least squares discriminant analysis (PLS-DA). (A) PLS-DA confirmed the distinct clustering of metabolites among the three groups. (B) Loading plot of the metabolites. [file 12967_2021_3129_MOESM1_ESM.pdf]
