# Supplementary material for: Hydrogen gas with extracorporeal cardiopulmonary resuscitation improves survival after prolonged cardiac arrest in rats
Source: J Transl Med. 2021 Nov 16;19:462. doi: 10.1186/s12967-021-03129-1 (PMC8594155; doi:10.1186/s12967-021-03129-1)
Supplement: Supplementary file 2 — Additional file 2: Table S1. Group characteristics before resuscitation in rats of placebo and H2 group. Table S2. Physiological and interventional variables during ECPR in rats of placebo and H2 group. Table S3. Detailed information of perturbed metabolites (VIP > 1.0) among the groups (Placebo vs. Pre-CA, or H2 vs. Placebo). Table S4. Result from metabolite set enrichment analysis (MSEA). [file 12967_2021_3129_MOESM2_ESM.pdf]

Table S1. Group characteristics before resuscitation in rats of placebo and H_2 group.

	Placebo (n=9)	H_2 (n=9)
Weight, g	449.6 ± 8.9	462.7 ± 8.4
HR at baseline, bpm	297 ± 8	283 ± 7
MAP at baseline, mmHg	64 ± 4	58 ± 2
Central venous pressure, mmHg	3.4 ± 0.8	1.7 ± 0.3
Teso, ˚C	37.1 ± 0.1	37.2 ± 0.1
$dp/dt_{(max)}$, mmHg/s	2360 ± 347	2486 ± 312
$dp/dt_{(min)}$, mmHg/s	-1142 ± 227	-1074 ± 134
$EtCO_2$ at baseline, mmHg	42.3 ± 1.5	43.1 ± 1.9
Time from vecuronium injection to CA, min	3.1 ± 0.1	2.8 ± 0.2

Values are mean ± SEM. HR, heart rate; MAP, mean arterial pressure; Teso, esophageal temperature; $EtCO_2$, End-Tidal carbon dioxide; CA, cardiac arrest. There was no significant difference in parameters between each group.

Table S2. Physiological and interventional variables during ECPR in rats of placebo and H_2 group.

	Placebo (n=9)	H_2 (n=9)
At 10 min after starting of ECPR		
HR, bpm	304 ± 11	313 ± 11
MAP, mmHg	63 ± 8	58 ± 4
Central venous pressure, mmHg	5.7 ± 0.8	3.1 ± 0.8 *
Pump flow rate, mL/min	61.3 ± 7.3	64.8 ± 3.7
At 30 min after starting of ECPR		
HR, bpm	353 ± 12	361 ± 18
MAP, mmHg	126 ± 8	110 ± 11
Central venous pressure, mmHg	3.6 ± 0.7	2.2 ± 0.8
Pump flow rate, mL/min	14.0 ± 3.4	14.0 ± 2.9
Time from ECPR initiation to ROSC, min	5.2 ± 1.2	3.7 ± 0.7
Duration of PEA, min	3.6 ± 1.1	2.1 ± 0.7

Values are mean ± SEM. HR, heart rate; MAP, mean arterial pressure; CPB, cardiopulmonary bypass; ROSC, return of spontaneous circulation; PEA, pulseless electrical activity. * P=0.036 vs placebo.

Table S3. Detailed information of perturbed metabolites (VIP>1.0) among the groups (Placebo vs. Pre-CA, or H2 vs. Placebo).

Metabolites	VIP	log2(FC) *	p value
**Placebo vs Pre-CA**			
D-mannitol	1.363	8.667	5.48E-14
myo-inositol	1.328	2.034	8.29E-10
uric acid	1.321	4.143	2.19E-09
citraconic acid	1.315	2.698	4.65E-09
phosphoric acid	1.310	1.699	8.51E-09
succinic acid	1.305	2.018	1.57E-08
allantoin	1.304	8.112	1.84E-08
2-hydroxybutyric acid	1.285	2.528	8.84E-08
glycerol 3-phosphate	1.281	2.651	1.38E-07
glycerol	1.273	1.963	2.30E-07
dehydroascorbic acid	1.262	3.714	4.78E-07
benzoic acid	1.256	-1.529	1.25E-06
hippuric acid	1.233	4.026	2.60E-06
maltose	1.221	3.881	4.21E-06
D-threitol	1.192	3.488	1.29E-05
citrulline	1.191	-1.835	1.50E-05
ethanolamine	1.183	2.593	1.97E-05
gluconic acid	1.139	0.965	7.67E-05
L-ornithine	1.129	1.172	1.15E-04

L-tryptophan	1.125	-0.909	2.29E-04
1-Methylnicotinamide	1.123	2.051	1.08E-04
psicose	1.118	2.496	1.35E-04
lactamide	1.100	1.830	1.89E-04
L-glutamic acid (dehydrated)	1.099	0.980	1.92E-04
3-methyl-2-oxobutanoic acid	1.077	2.129	3.56E-04
phosphoethanolamine	1.074	2.060	3.49E-04
Inosine	1.017	-2.944	1.21E-03
H2 vs Placebo			
myo-inositol	2.162	0.638	3.03E-03
L-histidine	1.964	-0.963	1.82E-02
raffinose	1.827	1.013	1.73E-02
D-mannitol	1.694	0.275	3.06E-02
L-glutamic acid	1.646	-1.035	3.63E-02
n-acetylneuraminic acid	1.633	1.379	4.25E-02

* FC = Placebo/Pre-CA or H2/Placebo.

Table S4. Result from metabolite set enrichment analysis (MSEA).

	total	expected	hits	Raw p	Holm p	FDR
D-Glutamine and D-glutamate metabolism	6	0.02	2	1.93E-04	1.62E-02	1.62E-02
Histidine metabolism	16	0.06	2	1.52E-03	1.26E-01	6.37E-02
Galactose metabolism	27	0.11	2	4.35E-03	3.57E-01	1.22E-01
Aminoacyl-tRNA biosynthesis	48	0.19	2	1.35E-02	1.00E+00	2.83E-01
Nitrogen metabolism	6	0.02	1	2.35E-02	1.00E+00	3.94E-01
Ascorbate and aldarate metabolism	8	0.03	1	3.12E-02	1.00E+00	4.36E-01
Arginine biosynthesis	14	0.06	1	5.40E-02	1.00E+00	6.07E-01
Butanoate metabolism	15	0.06	1	5.78E-02	1.00E+00	6.07E-01
beta-Alanine metabolism	21	0.08	1	8.01E-02	1.00E+00	6.71E-01
Alanine, aspartate and glutamate metabolism	28	0.11	1	1.06E-01	1.00E+00	6.71E-01
Glutathione metabolism	28	0.11	1	1.06E-01	1.00E+00	6.71E-01
Phosphatidylinositol signaling system	28	0.11	1	1.06E-01	1.00E+00	6.71E-01
Inositol phosphate metabolism	30	0.12	1	1.13E-01	1.00E+00	6.71E-01
Porphyrin and chlorophyll metabolism	30	0.12	1	1.13E-01	1.00E+00	6.71E-01
Glyoxylate and dicarboxylate metabolism	32	0.13	1	1.20E-01	1.00E+00	6.71E-01
Amino sugar and nucleotide sugar metabolism	37	0.15	1	1.37E-01	1.00E+00	6.97E-01
Arginine and proline metabolism	38	0.15	1	1.41E-01	1.00E+00	6.97E-01
